# Surveillance of Human Adenovirus Types and the Impact of the COVID-19 Pandemic on Reporting — United States, 2017–2023

**DOI:** 10.15585/mmwr.mm7350a1

**Published:** 2024-12-19

**Authors:** Fatima Abdirizak, Amber K. Winn, Rishika Parikh, Heather M. Scobie, Xiaoyan Lu, Everardo Vega, Olivia Almendares, Hannah L. Kirking, Erica Billig Rose, Benjamin J. Silk

**Affiliations:** 1 Coronavirus and Other Respiratory Viruses Division, National Center for Immunization and Respiratory Diseases, CDC.

**Summary**

**What is already known about this topic?**

Human adenoviruses (HAdVs) cause a wide spectrum of respiratory and nonrespiratory illnesses and are associated with outbreaks in congregate settings. The National Adenovirus Type Reporting System (NATRS) was formally initiated in 2014, but has conducted passive, voluntary surveillance of circulating HAdV types in the United States since 2003.

**What is added by this report?**

Reporting to NATRS decreased during and after the COVID-19 pandemic, despite continued HAdV circulation reported through passive laboratory surveillance to the National Respiratory and Enteric Virus Surveillance System. During 2017–2023, six HAdV types (1–4, 7, and 14) accounted for 88.3% of typed specimens; 17.0% of specimens were identified as outbreak-related.

**What are the implications for public health practice?**

Strengthened public health and clinical laboratory capacity to type and report HAdV-positive specimens is needed to improve understanding of HAdV type circulation patterns, which can guide outbreak investigations and support development of diagnostic tests, therapeutics, and vaccines.

## Abstract

Human adenoviruses (HAdVs) are typically associated with mild respiratory illnesses, although severe disease and outbreaks in congregate settings occur. The National Adenovirus Type Reporting System (NATRS) is a passive, laboratory-based surveillance system that monitors trends in circulation of HAdV types in the United States. This report summarizes the distribution of HAdV types reported to NATRS during 2017–2023. During this 7-year period, 2,241 HAdV specimens with typing results were reported to NATRS. The number of specimens with HAdV typing results reported varied annually during 2017–2019 (range = 389–562) and declined during 2020–2023 (range = 58–356). During 2017–2023, six HAdV types (1–4, 7, and 14) accounted for 88.3% of typed specimens reported; 17.0% of specimens were identified as outbreak-related. An increase in type 41 reporting was associated with a hepatitis cluster during 2021–2022. Reporting to NATRS has declined since the COVID-19 pandemic, despite continued HAdV circulation reported through passive laboratory surveillance to the National Respiratory and Enteric Virus Surveillance System. Enhanced participation in NATRS is needed to improve monitoring of circulating HAdV types.

## Introduction

Human adenoviruses (HAdVs) are classified into seven species (designated A–G) and approximately 100 types (designated with integers) (*1*). HAdV types are associated with various respiratory illnesses as well as nonrespiratory illnesses including gastroenteritis and conjunctivitis (*1*). HAdV types are sometimes associated with differing degrees of illness severity, although how HAdV type and host factors interact and result in a clinical syndrome is not fully understood. Most HAdV infections are mild or asymptomatic. However, severe illness can occur, either sporadically in otherwise healthy persons or, more frequently, in persons with immunocompromising conditions (*1*). Outbreaks of HAdV have been described in congregate settings including college campuses, hospitals, and military training sites (*1*–*4*). An HAdV vaccine against types 4 and 7 is only available for military personnel*; these types have caused outbreaks of severe respiratory illness. This report summarizes HAdV typing data reported during 2017–2023 to the National Adenovirus Type Reporting System (NATRS),^†^ a passive, laboratory-based surveillance system that monitors HAdV types across the United States.

## Methods

### Data Sources

Participating laboratories with HAdV typing results voluntarily report quarterly to NATRS. Results are accompanied by limited demographic, clinical, and laboratory data, and indication of whether the specimen was associated with an outbreak or cluster. Additional outbreak-related specimens are identified through manual review and follow-up communications with public health officials. Laboratory testing for HAdVs is performed by molecular assay, antigen detection, or virus isolation. The HAdV target is included in many commercially available polymerase chain reaction (PCR) panels for respiratory pathogens. Because HAdV testing and typing do not typically influence clinical management of patients with HAdV infections, diagnostic HAdV testing is not routine and few laboratories routinely perform HAdV typing.^§^ However, during public health investigations, such as those related to outbreaks, laboratory specimens are more likely to be sent for HAdV testing and typing to help characterize illnesses, determine the scope of the outbreak, and design interventions. Thus, outbreak specimens might be more likely to be reported to NATRS.^¶^

### Data Analysis

This report describes the distribution of HAdV results reported to NATRS during 2017–2023 by patients’ state of residence and age group. In addition, the distribution of typing results by specimen type and year of specimen collection were examined, and the HAdV types associated with outbreaks during the study period were identified. Reports with missing HAdV typing data, missing date of collection, and known instances in which multiple specimens of the same HAdV type were collected from the same patient within 90 days were excluded. This activity was reviewed by CDC, deemed not research, and was conducted consistent with applicable federal law and CDC policy.**

## Results

### Reports Submitted to NATRS

**FIGURE 1:**
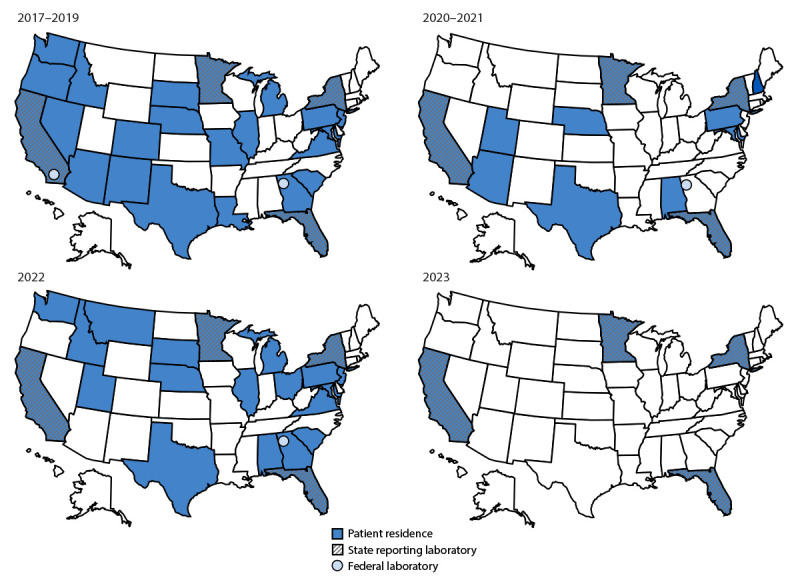
Geographic distribution of cases with human adenovirus typing data, by patient state of residence and reporting laboratory — National Adenovirus Type Reporting System, United States, 2017–2023*^,^^†^ **Abbreviations:** HAdV = human adenovirus; HHS = U.S. Department of Health and Human Services; NATRS = National Adenovirus Type Reporting System. * Typing results were reported to NATRS from the California Department of Public Health Laboratory (2017–2023); Coronavirus and Other Respiratory Viruses Diagnostic Laboratory, CDC (2017–2019 and 2021–2022); Bureau of Public Health Laboratories, Florida Department of Health (2017–2023); Public Health Laboratory, Minnesota Department of Health (2017–2023); Naval Health Research Center (2017–2019); and Wadsworth Center, New York State Department of Health (2017–2019 and 2021–2023). ^†^ Although the laboratories participating in NATRS are physically located in HHS Regions 2 (New York), 4 (Georgia and Florida), 5 (Minnesota), and 9 (California) only, specimens originated from patients living in 30 states and all 10 HHS regions.

Six laboratories reported results to NATRS for 2,909 specimens collected during 2017–2023, including CDC’s Respiratory Virus Diagnostics Laboratory, four state public health laboratories, and one U.S. Department of Defense laboratory. Specimens originated from patients living in 30 states (Figure 1). After exclusion of reports with missing HAdV typing data (381) or date of collection (274) and multiple specimens of the same HAdV type from the same patient (13),^††^ a total of 2,241 typing results were included in the analysis (Supplementary Table, https://stacks.cdc.gov/view/cdc/168891).

**FIGURE 2:**
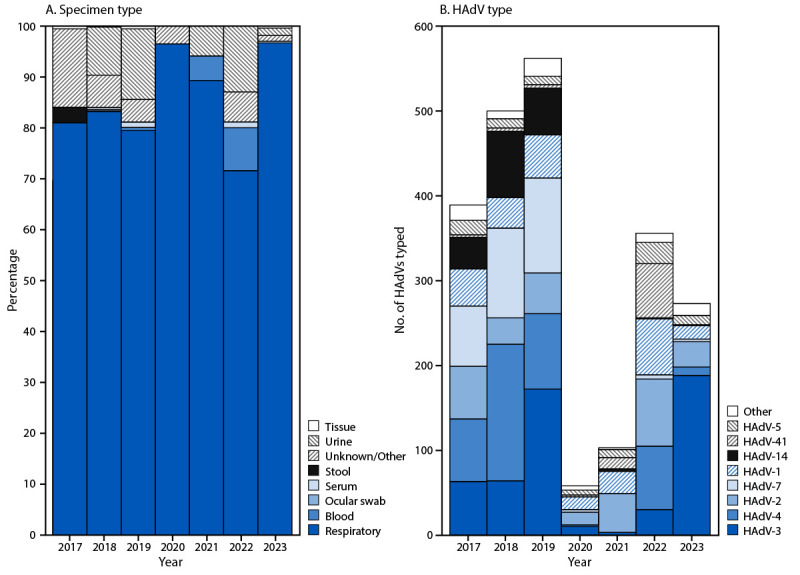
Percent distribution of specimen types reported (A) and number of human adenovirus types (B),* by year of specimen collection — National Adenovirus Type Reporting System, United States, 2017–2023 **Abbreviation:** HAdV = human adenovirus. * Frequencies of the top three HAdV types each year are as follows: 2017: HAdV-4 (19.0%), HAdV-7 (18.3%), and HAdV-3 (16.2%); 2018: HAdV-4 (32.2%), HAdV-7 (21.2%), and HAdV-14 (15.6%); 2019: HAdV-3 (30.6%), HAdV-7 (19.9%), and HAdV-4 (15.8%); 2020: HAdV-2 (25.9%), HAdV-1 (25.9%), and HAdV-3 (17.2%); 2021: HAdV-2 (44.7%), HAdV-1 (25.2%), and HAdV-41 (12.6%); 2022: HAdV-2 (22.2%), HAdV-4 (21.1%), and HAdV-1 (18.5%); 2023: HAdV-3 (68.9%), HAdV-2 (11.0%), and HAdV-1 (5.9%).

Respiratory specimens accounted for the largest percentage of specimens (82.3%); the second largest group included stool specimens, which accounted for 6.4% of all specimens (Table). Each year, respiratory specimens constituted >70% of reported types; during 2017, 2018, and 2022, stool represented >5% of specimens (Figure 2).^§§^

**TABLE:** Distribution of human adenovirus types, specimen types, and outbreak-related cases — National Adenovirus Type Reporting System, United States, 2017–2023

HAdV type	No. (column %)	Specimen type, no. (row %)			Outbreak-related cases no., (row %)
		Respiratory	Stool	Other*	
3	530 (23.7)	493 (93.0)	5 (0.9)	32 (6.0)	9 (1.7)
4	411 (18.3)	327 (79.6)	65 (15.8)	19 (4.6)	141 (34.3)
2	311 (13.9)	278 (89.4)	5 (1.6)	28 (9.0)	7 (2.3)
7	300 (13.4)	258 (86.0)	22 (7.3)	20 (6.7)	86 (28.7)
1	254 (11.3)	219 (86.2)	7 (2.8)	28 (11.0)	8 (3.1)
14	174 (7.8)	128 (73.6)	10 (5.7)	36 (20.7)	75 (43.1)
41	91 (4.1)	21 (23.1)	27 (29.7)	43 (47.3)	39 (42.9)
5	90 (4.0)	73 (81.1)	0 (—)	17 (18.9)	2 (2.2)
Other^†^	80 (3.6)	48 (60.0)	2 (2.5)	30 (37.5)	14 (17.5)
**Total (row %)**	**2,241 (100.1)^§^**	**1,845 (82.3)**	**143 (6.4)**	**253 (11.3)**	**381 (17.0)**

**Abbreviation:** HAdV = human adenovirus.

* Other specimen type includes unidentified source (183; 72.3%), blood (39; 15.4%), ocular swab (13; 5.1%), serum (13; 5.1%), urine (4; 1.6%), and tissue (1; 0.4%).

^†^ Other HAdV types include 6, 8, 9, 11, 12, 21, 22, 25, 31, 35, 37, 40, 53, and 56.

^§^ Total percentage does not sum to 100.0 because of rounding.

Annual reporting to NATRS increased from 389 specimens in 2017 to 562 in 2019. However, annual reports declined during the COVID-19 pandemic in 2020 (58 specimens) and 2021 (103). In 2022 and 2023, a total of 356 and 273 reports, respectively, were received, lower than the average annual mean of 484 reported during 2017–2019 (Figure 2). Throughout the study period, specimens with typing results reported to NATRS were most frequently collected from infants and young children aged 0–4 years (1,009; 45.0%), young adults aged 18–29 years (616; 27.5%), and children and adolescents aged 5–17 years (359; 16.0%). Typed specimens collected from adults aged ≥30 years were infrequent (95; 4.2%); patient age was missing for 162 (7.2%) specimens.

During 2017–2023, six HAdV types accounted for 88.3% of all reported data: 530 (23.7%) type 3; 411 (18.3%) type 4; 311 (13.9%) type 2; 300 (13.4%) type 7; 254 (11.3%) type 1; and 174 (7.8%) type 14. The annual distribution of HAdV types varied (Figure 2). During 2017–2019, types 4 (22.3%), 3 (20.6%), and 7 (19.9%) were the most frequently reported; however, during 2020–2022, types 2 (27.1%), 1 (20.7%), 41 (15.3%), and 4 (14.9%) were most frequently reported. In 2023, types 3 (68.9%) and 2 (11.0%) predominated.

Among all 2,241 specimens reported during 2017–2023, a total of 381 (17.0%) were identified as outbreak-related (Table). Among 1,451 typed specimens reported during 2017–2019, a total of 267 (18.4%) were outbreak-related. Among these, the most frequent outbreak-related types were 7 (32.2%), 4 (29.2%), and 14 (27.0%). During 2020–2021, among 161 specimens, 17 (10.6%) were related to an outbreak, including a nationwide investigation into pediatric hepatitis suspected to be associated with type 41 adenovirus infection.^¶¶^ Among these specimens, 11 (64.7%) were type 41. In 2022, among 356 specimens reported, 97 (27.2%) were outbreak-related; most were types 4 (65.0%) and 41 (26.8%). No outbreak-related specimens were identified in 2023.

## Discussion

HAdV type surveillance is useful for monitoring circulation patterns over time, guiding outbreak investigations, and supporting development of diagnostic tests, therapeutics, and vaccines. During 2017–2023, the most frequently reported HAdV types (1–4, 7, and 14) accounted for 88% of NATRS specimens. Similarly, a previous NATRS report found that these same six types accounted for 86% of specimens reported in the United States during 2003–2016 (*5*). These surveillance findings suggest that vaccines and therapeutics with coverage of certain HAdV types could help mitigate community and outbreak-related illness.

Because HAdV typing does not typically influence clinical management, a substantial proportion (17%) of typing results reported to NATRS were identified as being associated with an outbreak or other epidemiologic investigation. During 2021–2022, type 41 was more frequently reported, largely because of the enhanced investigations of HAdV-associated acute pediatric hepatitis (*6*,*7*). Outbreaks in congregate settings such as colleges, health care facilities, and military environments, particularly those involving HAdV types 4 and 7, influenced the observed trends in HAdV type distribution during the study period. In 2017, an outbreak associated with HAdV type 7 occurred among unvaccinated military trainees in Virginia (*2*), and in 2019, an outbreak associated with type 4 occurred at a military training academy in Connecticut (*4*). During 2018–2022, types 4 and 7 were linked to multiple college outbreaks across several states (*3*,*8*,*9*).

NATRS reporting has declined substantially since the COVID-19 pandemic. Implementation of nonpharmaceutical interventions in response to the pandemic in 2020 disrupted the circulation of most respiratory viruses (*10*). As a result, respiratory HAdV circulation reported to the National Respiratory and Enteric Virus Surveillance System (NREVSS), a passive, laboratory-based surveillance network, initially decreased that year. However, by spring 2021, HAdV circulation reported to NREVSS had returned to typical prepandemic levels, with elevated activity observed in fall 2022 followed by a return to typical prepandemic circulation by fall 2023.*** Thus, the decline in NATRS participation during the pandemic is most likely related to reduced HAdV typing or reporting of HAdV types rather than to diminished HAdV activity.

### Limitations

The findings in this report are subject to at least four limitations. First, a limited number of laboratories have HAdV typing capacity; thus, data reported to NATRS are not geographically representative of HAdV circulation. Second, reports with missing typing or date of collection data were excluded, further limiting the representativeness of the data. Third, data reported to NATRS do not include detailed epidemiologic or clinical information; therefore, HAdV types could not be associated with specific clinical syndromes or outcomes. Finally, HAdV typing is differentially performed on specimens from severe infections and outbreaks; therefore, NATRS results are unlikely to represent the full distribution of HAdV types circulating in the community.

### Implications for Public Health Practice

NATRS is designed for national tracking of circulating HAdV types. However, reporting has declined since public health resources were diverted to the COVID-19 pandemic. To enhance national surveillance and geographic representativeness, laboratories are encouraged to perform typing or refer positive specimens for typing and report their results to NATRS.^†††^ Increasing routine typing of HAdV from both community and outbreak sources is essential for detecting outbreaks locally as well as understanding differences in circulating genotypes and changes in epidemiology and clinical severity. The expanded application of pathogen genomics (i.e., whole genome sequencing) and associated resources since the pandemic can be further leveraged to improve HAdV type surveillance. Expanded laboratory capacity to type HAdVs and enhanced reporting could improve national understanding of HAdV circulation patterns and better guide public health prevention strategies.

## Footnotes

*
https://www.cdc.gov/vaccines/hcp/vis/vis-statements/adenovirus.html

†
https://www.cdc.gov/adenovirus/php/surveillance/about-natrs.html

§ Type identification of HAdV-positive specimens requires additional testing. Because relatively few clinical or public health laboratories currently perform HAdV typing, those with typing capacity (including the CDC diagnostic laboratory) have provided technical assistance during epidemiologic investigations or outbreaks.

¶ Laboratory guidance and resources for adenovirus typing are provided by CDC and the Association of Public Health Laboratories. https://www.cdc.gov/adenovirus/php/laboratory-testing/index.html; https://www.aphl.org/programs/infectious_disease/Pages/Adenovirus.aspx

** 45 C.F.R. part 46, 21 C.F.R. part 56; 42 U.S.C. Sect. 241(d); 5 U.S.C. Sect. 552a; 44 U.S.C. Sect. 3501 et seq.

†† No cases of the same HAdV type from the same patient but from different sources were found.

§§ During the 7-year study period, molecular typing methods, including partial or full genome sequencing (1,327; 59.2%) and type-specific real-time PCR (756; 33.7%), were predominantly used for HAdV typing. The traditional serum neutralization test was used infrequently (103; 4.6%).

¶¶ The HAdV type 41 cases were not associated with a single outbreak but were part of a nationwide epidemiologic investigation during 2021–2022 that resulted in increased reporting of acute hepatitis associated with HAdV infection among children.

*** NREVSS collects the aggregate weekly number of tests performed and the total number of those tests that were positive from participating U.S. laboratories (https://www.cdc.gov/nrevss/php/dashboard/index.html). Among 1,799,424 HAdV tests reported to NREVSS during 2017–2019, there were 54,782 HAdV detections; among 2,908,127 HAdV tests reported during 2021–2023, a total of 120,928 HAdV detections were reported. NREVSS does not collect data on HAdV type.

††† Laboratories with HAdV typing results can report data to NATRS at natrs@cdc.gov.
